# Study protocol of a quasi-experimental trial to compare two models of home care for older people in the primary setting

**DOI:** 10.1186/s12877-020-1497-0

**Published:** 2020-03-12

**Authors:** Carolina Burgos-Díez, Rosa Maria Sequera-Requero, Francisco José Tarazona-Santabalbina, Joan Carles Contel-Segura, Marià Monzó-Planella, Sebastià Josep Santaeugènia-González

**Affiliations:** 1 Department of Surgery and Surgical Specializations, Faculty of Medicine, University of Barcelona (PC 08036), Barcelona, Catalonia Spain; 2 Primary Care Center Apenins, Badalona Serveis Assistencials, Badalona, Catalonia Spain; 3 Primary Care Center Gran Sol, Institut Català de la Salut, Badalona, Catalonia Spain; 4 Geriatric Medicine Department, Hospital Universitario de la Ribera, Alzira, Valencia Spain; 5 Chronic Care Program, Ministry of Health, Barcelona, Catalonia Spain; 6 Central Catalonia Chronicity Research Group (C3RG), Centre for Health and Societal Care Research (CESS), University of Vic - Central University of Catalonia (UVIC-UCC), C. Miquel Martí i Pol, 1, 08500 Vic, Spain

**Keywords:** Home care models, Preventive home visits, Primary care, Geriatric assessment

## Abstract

**Background:**

Preventive home visits are suited for patients with reduced mobility, such as older people. Healthcare needs for older patients are expected to increase due to the extended life expectancy estimated in coming years. The implementation of low-cost, patient-centered methodologies may buffer this rise in health care costs without affecting the quality of service. In order to find the best home care model with less investment, this paper describes a study protocol comparing two models of home care for older people.

**Methods:**

We describe a quasi-experimental study that compares the outcome of two different home care models already implemented in two primary care centers in Badalona (Barcelona, Spain). The traditional model (control model) is integrated in the sense that is continuous, the same primary care center team looks after its assigned patients both at the center and in preventive home visits. The new functional home care model (study model), consisting of a highly trained team, is specifically designed to meet patient needs and give total attention to preventive home interventions. The study will start and end on the expected dates, June 2018 to October 2020, and include all patients over 65 years old already enrolled in the home care programs of the primary care centers selected. The primary endpoint assessed will be the difference in hospitalization days between patients included in both home care programs. Other variables regarding health status, quality of care and resource utilization will also be compared between the two models.

**Discussion:**

The study in progress will assess whether a functional and highly trained home care team will meet the ever-aging population needs in terms of cost and health outcomes better than a traditional, integrated one. Lessons learned from this pilot study will provide guidelines for a future model of home care based on the IHI Triple Aim: better care, better health, and lower costs.

**Trial registration:**

Registered in ClinicalTrials.gov (Identifier: NCT03461315; March 12, 2018).

## Background

The increasing life expectancy in industrialized countries correlates with a rising prevalence of chronicity, disability, and dependence, thus stressing the need for healthcare services to make progress [1, 2]. In Spain, multimorbid patients managed in a primary care setting account for 1.38% of the overall population and 5% of people older than 65 years old [3]. The increasing trend of these percentages will result in a greater use of health care resources, including medical appointments, visits to the emergency service, hospitalization, and medication [1–4]. Therefore, it is necessary to find cost-effective strategies to cover the healthcare needs of older patients.

In the last decade, various approaches to care delivery for older people have been proposed, yielding a repertoire of models that range from traditional primary care and total patient models to integrated care models [5–7]. As part of the services offered, home care (HC) programs are intended to provide health care to patients that are unable to go to a primary care center due to their medical condition or disability, thus improving their health and active independence and reducing hospitalizations [8–11]. Some of these HC programs prioritize continuity in the sense that the same medical professional team (i.e., physician and nurse) attends the patient both at home and at the primary care center. This model, often referred to as integrated HC, has the advantage of strengthening the relationship between patients and their healthcare provider but struggles with the staff’s overloaded schedule [12, 13]. Other extensively applied models are based on skilled HC teams dedicated solely to at-home interventions; this approach, known as functional HC, increases specialization of the HC team and reduces the overburden of health care professionals, but may hamper the cohesion of healthcare services [14–16]. These models were implemented with positive outcomes regarding patients’ quality of life and care delivery satisfaction. However, studies investigating the outcome of HC have yielded heterogenous results, which likely depend on multiple factors including population factors, program characteristics, and healthcare setting [15–17]. In fact, even healthcare environments were HC is well established as a reality for care of older and disabled people acknowledge that more in-depth analysis is needed to improve understanding of the role of HC in the future of health care delivery [18]. Taken together, this heterogeneity precludes the appraisal of the benefits and drawbacks of HC approach, often leading to inconclusive evidence [19].

Catalonia is the most north-eastern region of Spain, with a population of over 7.5 million people, 19% of which is older than 65 years. Here, HC programs are highly implemented, with nearly 90% of primary care units using traditionally integrated HC approaches within each provision of healthcare service, that enroll a 5% of the population older than 65 years. However, a survey of primary care professionals involved in HC programs revealed important disadvantages of this model, such as a high burden of care, time constraints, low resources and lack of coordination with other levels of care as each of the services provides HC assistance besides their center-based duties [20]. The change from a traditional integrated HC model to a functional full-time HC is expected to result in better outcomes for patients (clinical and related to the attendance experience), increased satisfaction for the professionals involved, and less use of healthcare resources. However, there is little or no evidence regarding the benefits and drawbacks of the two models when operating in the same healthcare environment. This study aims to compare two HC programs for older people applied in Catalonia: a traditional integrated HC model provided by healthcare professionals of primary care units and a specific full-time unit of medical professionals based on a functional HC model.

## Methods

### Study design

This quasi-experimental study will compare the outcome of two HC models implemented in two primary care centers in Badalona (Barcelona, Spain). The control group will consist of patients following the integrated HC model, provided by the primary care center *Gran Sol*, and the study group will consist of patients following the new HC model (hereinafter, functional HC model), linked to the primary care center *Apenins*. Both HC teams consist of a general practitioner and a nurse. In the integrated model, HC is given by the same healthcare team providing medical care at the primary care unit with an average of 1500 inhabitants assigned to each team. By contrast, in the functional model, HC is given by a healthcare team specifically trained in the management of older, frail and multimorbid patients, providing only full-time preventive home visits and connecting to other further required special provision services. Further details regarding the characteristics of each model are shown in Table 1. The rationale for choosing these two centers relates to the balanced demographic characteristics of the reference population (Table 2).

**Table 1:** Main characteristics of the two investigated models

Integrated HC		Functional HC
Characteristics of the healthcare team		
**Team composition**	Nurse and family doctor.	
**Team function**	The same healthcare team provides HC and manages patients in the primary care center.	The healthcare team is dedicated exclusively to HC.
**Interprofessional communication**	Healthcare professionals are part of the healthcare team regularly managing patients in the primary care center.	Although not managing patients in the primary care center, the HC team is part of the health care staff of the center and their members participate in the center meetings as specialists.
**Training**	• Regular training of family doctors, including regular stays at mental health and geriatrics units. • Regular training of nurses.	• Regular training of family doctors, including regular stays at mental health and geriatrics units. • Nursing staff receives additional training regarding the management of chronic patients, fragility, and palliative care.
**Type of professional in each visit**	Nurse, family doctor or both	
Services and visit schedule		
**Preventive visits**	• Visits of nursing staff scheduled based on the monitoring requirements of each disease as established by local guidelines. • Visits of physician scheduled at physician’s discretion based on the disease progression and clinical status of patients.	
**Non-urgent acute visits**	The patient calls the center and the physician available at that moment (not always the one regularly visiting the patient at the primary care center) visits the patient at home.	• During working hours: the patient contacts directly the physician of the HC team. • Outside the working hours: the patient calls the center and the physician available at that moment (not always the one regularly visiting the patient at the primary care center) visits the patient at home.
**Urgent visits**	The patient calls the emergency service; an emergency team and an ambulance are deployed to patient’s home.	
**Financial approach**	All visits are fully covered by the public health system.	

*HC* home care

**Table 2:** Characteristics of the participating centers

	Integrated HC (PC Gran Sol)	Functional HC (PC Apenins)	***P***^a^
Location	Badalona, Catalonia, Spain	Badalona, Catalonia, Spain	
Professional profile	MDs and nurses specialized in family medicine	MDs and nurses specialized in family medicine	
Reference population^b^, *No.*	19,442	19,043	
Over-Aging index^c^, *%*	11%	9.2%	< 0.001
Foreign population^d^, *n (%)*	3499 (17.9%)	3046 (15.9%)	< 0.001
≥65 years old, *n (%)*	3480 (17.9%)	2970 (15.6%)	< 0.001
GMA, *adjusted indicator (IC95%)*	1.189 (1.173–1.206)	1.178 (1.161–1.195)	–
Mortality, *annual ‰*	7	5.7	0.143
IT application	eCAP	eCAP	

*HC* Home Care, *GMA* Adjusted Morbidity Groups [21]; *IT* Information Technology, *MD* Medical Doctor, *PC* Primary Care

^a^Differences between PC Gran Sol and PC Apenins

^b^Data from Msiq (Generalitat de Catalunya©), period between January and December 2015

^c^The number of persons aged 74 or over per total of persons over 64 years old

^d^The number of subjects with a foreign nationality

**Fig. 1:**
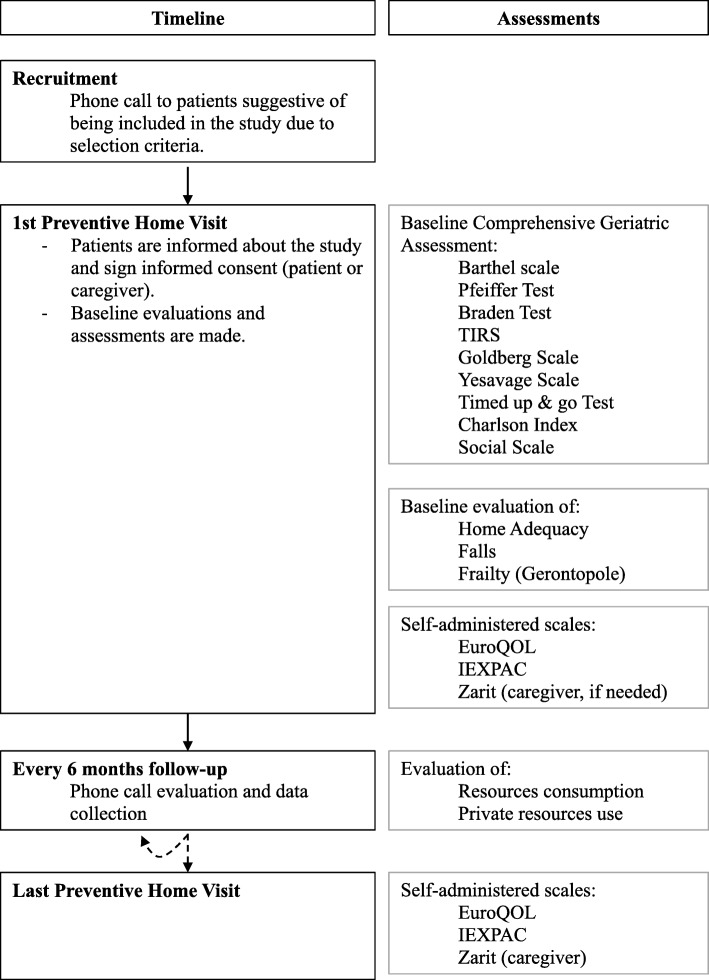
Flowchart of interventions to patients during the study

After signing the consent form to participate in the study during a preventive home visit, patients (or their caregivers) are provided with a set of self-administered questionnaires and scales, as well as a return envelope. In the case of patients with cognitive impairment, the documents are delivered to the caregiver. Investigators will contact study patients by phone every 6 months and ask them about their admissions to other centers than the assigned primary care center. At the end of follow-up (i.e., 2 years), study patients (or their caregivers) will be provided with the same self-administered questionnaires and scales given at baseline in a final preventive home visit (Fig. 1). Clinical and demographic data will be retrieved from patients’ medical records. All the information retrieved from the three sources (i.e., questionnaires/scales, check-up phone calls, and medical records) will be recorded in a clinical report form specifically designed for the study. Before the baseline visit, all investigators will participate in two training sessions on the scales and questionnaires used in the study.

All the HC interventions performed on both HC programs are based on current protocols designed following recommendations of the semFYC (Spanish Society of Family and Community medicine) and EUROPREV (European Network for Prevention and Health Promotion in Family Medicine and General Practice) inside the Program of preventive activities for Health promotion (PAPPS) [22]. The study protocol was approved by the IDIAP Ethics Committee of the Jordi Gol Foundation (Approval code: P17/121). Patients will volunteer and sign an informed consent, and all the information gathered will be anonymized before conducting any analysis. All data will be handled according to the Spanish Data Protection Law (LOPD) 15/1999 and the EU General Data Protection Regulation 2016/679.Considering the routine interventions defined in the study, conventional risks are not expected to increase. The last visit of the last patient recruited is foreseen by October 2020.

### Selection criteria

All patients aged over 65 years old and enrolled in the long-term HC program at any of the two participating primary care centers for at least 3 months will be considered for eligibility. Patients will be included irrespective of their cognitive status. Exclusion criteria include patients with a life expectancy of less than a month and patients with a score of 5 or more in the Pfeiffer’s test that does not have a full-time caregiver or that have a part-time one because a severe cognitive impairment is likely to interfere with the study procedure. Patients that are not registered as Badalona citizens will also be excluded because it is assumed they have temporary status, as well as patients included in a HC program due to their reduced mobility to reduce bias when measuring patient-requested interventions because they cannot easily reach the primary care center facility.

### Patient recruitment

Between June and October 2018, all subjects included in the HC program at the two primary care centers that met the selection criteria were contacted by phone and offered to participate in the study. Patients willing to do so were scheduled a domiciliary visit to receive the study documents (i.e., the Patient Information Sheet and self-administered questionnaires/scales) and sign the informed consent themselves or, in case of cognitive impairment, their full-time caregivers.

### Study conduct

The study started in June 2018 and is expected to end by October 2020. On the first preventive home visit, once informed consents have been accepted, patients, or the caregivers in case of cognitive impairment (defined as subjects scoring 5 or more in the Pfeiffer’s test), will be given self-administered questionnaires like EuroQoL, IEXPAC, and Zarit [23–25]. All of them will be analyzed by the investigator, who will assess the patient’s frailty in situ. The self- administered scales will be completed again by the patient and/or caregiver 2 years later, in a preventive home visit at the end of the study. Besides this start and end visits, participants will be interviewed by phone every 6 months to solve any issues and find out if any private hospitalizations or daycare centers have been used. Also, all visits requested by either the patient or the reference doctor will be reported in a case report form (CRF). The medical professionals involved in the study performing the preventive home visits will be trained for the study and in the use of the scales to ensure consistency and reinforce their implication.

All data, irrespective of their source, will be recorded in an anonymized CRF, in which patients will be identified with a study code. The study investigator will keep a key table with the study codes and their corresponding medical record identification codes.

### Endpoints and variables

The primary endpoint will be the difference in mean days of hospital stay per year between patients included in the integrated and functional HC programs. Secondary endpoints will include the assessment of the differences between the two HC models i.e., mortality and hospital admissions, based on the IHI Triple Aim (Better Care, Better Health, Lower Costs) [26]. To this end, variables regarding subjects’ health status, quality of care, and resource utilization of patients included in the two models will be compared (Table 3). The demographic characteristics of the study participants will also be recorded.

**Table 3:** Study variables

	Definition and Measurements	Categories
Demographic Characteristics		
Age	At the moment of entering the HC program	65–74 75–84 > 85
Gender		Male | Female
Health Status		
Caregiver	At the time of entering the HC program	Yes | No
Level of dependency	Scale defined by Royal Decree 174/2011; Law 39/2006 of Promotion of Personal Autonomy and Care for People in Situations of Dependency.	Grade I (25–49): Moderate dependency Grade II (50–74): Severe dependency Grade III (75–100): Great dependency
Decubitus ulcer	At physician’s discretion	Presence | absence
Drugs prescribed	By active substance.	Not polymedicated (< 5) Polymedicated (5–10) Hyperpolymedicated (> 10)
Comorbidity burden	Measured by Adjusted Morbidity Groups (GMA) risk assessment tool. GMA considers the type of disease (i.e. acute or chronic), number of systems affected, and complexity of each disease, which is coded by the International Classification of Diseases (ICD-9-CM) and stratify depending on the complexity.	31 qualitative ordinal GMA levels.
Mortality	Death during follow-up	Yes | No
Realization of CGA		Yes | No
Assessments included in CGA		
Performance of normal daily tasks	Barthel Scale [27]	Total dependency (< 20) Severe dependency (20–60) Moderate dependency (61–90) Mild dependency (91–99) Autonomous (100)
Mental health	Pfeiffer’s Test [28]	High risk (≤2) Mild cognitive impairment (3–4) Moderate cognitive impairment (5–7) Severe cognitive impairment
Decubitus ulceration risk	Braden Test [29]	High risk (< 12) Moderate risk (13–14) Low risk (15–18)
Social risk	TIRS [30]	There is social risk when one indicator over 6 is positive
Anxiety	Goldberg Scale [31]	Probable anxiety (≥4 positive responses) Probable depression (≥2 positive responses)
Geriatric depression scale	Yesavage Scale [32]	No depression (1) Possible depression (≥ 2)
Mobility	Timed Up and Go Test [33]	Normal (< 10) Very little impaired (10–19) Moderately impaired (20–29) Severely impaired (≥30)
Comorbidity	Charlson Index [34]	No comorbidity (0–1) Low comorbidity burden (2) High comorbidity burden (> 3)
Social state	Social Scale [35]	No social risk (≤9) Social risk (10–14) Social problem (≥15)
Overburden of the caregiver	Zarit Test [25]	No overburden (< 46) Intense overburden (> 56)
Nutritional state evaluation	MNA [36]	Malnutrition (≤7) Malnutrition risk (8–11) Normal nutritional status (≥12)
Home adequacy	Revision of home adequacy to determine the need of social worker intervention	Social worker is recommended when at least one item is positive
Falls	Number of falls in the last year	–
Frailty	Gérontopôle Frailty Screening Tool [37]	Frailty identification if one item of the scale is positive.
Social services cost	Number and cost of social worker visits	–
Quality of life	EuroQOL 5D-3 L [24, 38, 39]	No problems (Level 1) Some problems (Level 2) Extreme problems (Level 3)
Quality of Care		
Physicians PQI	Prescription Quality Index of the physician [40]	Include assessment of three categories: More adequate therapeutic alternatives Hyper prescription of a particular group of drugs Selection indicators to promote safer and more effective alternatives available
Prescriptions	Number of prescription events per patient	–
Alternative drugs with therapeutic benefit	Percentage of use of the alternative drug as assessed by the physician	–
Shared Interdisciplinary Individual plan		Yes | No
Chronicity	Number of Complex Chronic Patients and Advanced Chronic Disease patients in the program	–
Satisfaction with health care received	IEXPAC Scale [23]	Satisfactory (=10) Unsatisfactory (< 10)
Overburden of the caregiver	Zarit Scale [25]	No overburden (< 46) Intense overburden (> 56)
Resource Utilization		
Family doctor visits	Number of at-home visits, other medical professional, primary care center or virtual appointments of the patient or the caregiver	–
Nursing staff visits	Number of at-home visits, other medical professional and primary care center appointments of the patient or the caregiver. Also, number of virtual consultations, including remote evaluation or by phone of medical record	–
Hospitalizations	Number of programmed, emergency and daycare admissions	–
Readmissions	Number of successive hospitalizations due to the same pathology in less than 30 days	–
Admissions to other public health care centers	Number of admissions, including convalescence, subacute, long-lasting and palliative units	–
Hospitalizations per year	Days hospitalized	–
Intermediate resources	Number and cost of laboratory tests, Radiology and interconsultations	–
Pharmaceutical costs	Expenditure per patient and medical professional during a year	–
Hospitalization-at-home device use	Number of activations per year and total of hospitalization days	–
Social services intensity	Monthly hours spent by the social worker. Costs derived from teleassistance, cleaning and cooking aid, reused orthopedic material and family worker	–
Call to emergency services	Number of patient intervention requests per year.	–
Perception of healthcare professionals	Qualitative, self-administered questionnaire of difficulties in HC practice, based on the survey reported by Linares et al. [20]	

*CGA* Comprehensive Geriatric Assessment, *HC* Home Care

Particularly, the baseline health status of study subjects will include the Gerontôpole frailty screening tool and the Adjusted Morbidity Groups (GMA) risk assessment tool, which considers the type of disease, number of systems affected, and complexity of each one [21, 37]. Additionally, a complete baseline Comprehensive Geriatric Assessment (CGA) will be performed, including the following assessments: the ability to perform normal daily tasks (Barthel scale), mental health status (Pfeiffer test), decubitus ulceration risk (Braden test), social risk (TRIS), geriatric depression (Yesavage scale), mobility status (Timed up & go test), comorbidity burden (Charlson Comorbidity Index), nutritional state (Mini-nutritional Assessment), and social status (Table 3) [27–35, 41]. The health-related quality of life of study participants and satisfaction of caregiver will be assessed at baseline and at the final follow-up visit using the EuroQoL IEXPAC and Zarit questionnaires, respectively [23–25].

### Statistical analysis

The sample size calculation was based on an incidence of hospital admission of 40% and a reduction of 10% in the study group, a 2-year follow-up, and a 1:1 ratio for control and intervention groups. Under these constrains, a fixed alpha and beta errors of 5%, yielded an estimated size of 581 subjects per group. The statistical power of this sample, assuming alpha and beta errors of 5% was 85.3%.

All collected variables will be described for the overall study sample and for both study groups. Quantitative variables will be described as the mean and standard deviation (SD), and as the median and interquartile range (IR) for normally- and non-normally distributed variables, as confirmed by the Kolmogorov-Smirnov test. Categorical variables will be described as frequencies and percentages. Measures of central tendency will be compared using the T-test for independent samples or ANOVA, or their non-parametric counterparts: the Mann-Whitney U and Kruskal-Wallis tests. Categorical variables will be compared using the Chi-square test or Fisher’s exact test. If post hoc analyses are performed, the Bonferroni or the Games-Howell adjustments will be applied. Variables with differences in the bivariate analysis at baseline (*p* < 0.1) and those considered clinically relevant for the authors will be included in a linear multiple logistic regression to build a multivariate model to predict the difference in mean days of hospital stay and costs of patients in the HC program. To address Better Care and Better Health endpoints, the authors will apply a binary logistic regression for mortality and hospital admission variables; age, gender, and comorbidity burden will be included as adjustment variables. A backward stepwise regression will be used to avoid overfitting of the model obtained.

A significance threshold will be set at two-sided alpha value < 0.05. The analysis will be performed with SPSS (IBM Corp. Released 2012. IBM SPSS Statistics for Windows, Version 21.0. Armonk, NY: IBM Corp.).

## Discussion

Since life expectancy of the population is increasing in developed countries, healthcare policymakers are prompted to tailor healthcare services to the specific needs of older people. In this context, the local healthcare authorities in Badalona (Spain) decided to change from a integrated HC model to a functional HC model, in which preventive home visits are provided by a dedicated healthcare team, different from that looking after patients in primary care units. Although various models of functional care have been proposed to prevent unnecessary hospitalizations and contribute to better health outcomes in post-acute patients [15, 16, 42], different populations and healthcare settings make it necessary to assess the outcome of this change in our area.

The main strengths of the functional model include a faster response to emergencies and the specialization of healthcare professionals. On the other hand, a potential disadvantage of this model —compared with the integrated one— is that it breaks the continuity of care (i.e., the patient is attended by a different healthcare team in the primary care unit and at home). Despite this limitation, the functional model is expected to yield better outcomes. In addition to the comparative analysis between both models, previous experiences in similar populations from our area will serve as a reference to interpret the results obtained [43, 44].

An important strength of this study is the homogeneity of both study groups. The quasi-experimental design precludes an active selection of the study sample, which is naturally defined by patients attending one of the primary care units based on their location. However, the characteristics of the reference population of both centers are very similar in terms of age and complexity (measured using the GMA risk [21, 45, 46]), suggesting that the baseline characteristics of study patients are likely to be balanced at the end of the study. Furthermore, unlike other change assessments in the HC model, this study will capture data prospectively, thus favoring the accuracy of the final record.

On the other hand, some limitations can be predicted. Firstly, a possible Hawthorne effect, according to which the quality of care may improve due to the fact that healthcare professionals are being assessed [47] cannot be ruled out. To our understanding, this effect —in case it occurs— is likely to decline throughout follow-up. Secondly, it seems almost unavoidable that patients will fail to report the use of private facilities, a fact that shall be considered at the moment of reporting results. Finally, this study has a risk of unbalanced reporting bias due to the differences in the healthcare teams of each model. Specifically, whereas in the integrated HC group multiple observers will contribute to data capture, in the functional group all assessments will be performed by a single HC team. In this regard, the reporting bias is likely to be attenuated in the integrated group by the presence of multiple observers, but not in the functional group. To increase homogeneity in practices and data recording, all investigators will receive a brief training.

In summary, the proposed study will permit assessment of health outcomes, feasibility, and resource utilization of new functional HC model and identify its strengths and weaknesses compared to the integrated model. The results obtained from this study may have important policy implications.

## Data Availability

Data sharing is not applicable to this article as no datasets were generated or analyzed during the development of this protocol. All data generated or analyzed during this study are included in this published article and its supplementary information file.

## Abbreviations

CGA: Comprehensive Geriatric Assessment
CRF: Case Report Form
GMA: Adjusted Morbidity Group
HC: Home Care
IEXPAC: Instrument to Evaluate the Experience of Patients with Chronic Diseases
IR: Interquartile Range
IT: Information Technology
MD: Medical Doctor
MNA: Mini Nutritional Assessment
PC: Primary Care
PQI: Prescription Quality Index
SD: Standard Deviation
TIRS: Test Indicators of Social Risk
